# Supplementary material for: Innovative design of minimal invasive biodegradable poly(glycerol-dodecanoate) nucleus pulposus scaffold with function regeneration
Source: Nat Commun. 2023 Jun 30;14:3865. doi: 10.1038/s41467-023-39604-0 (PMC10313828; doi:10.1038/s41467-023-39604-0)
Supplement: Supplementary file 1 — Supplementary Information [file 41467_2023_39604_MOESM1_ESM.pdf]

Supplementary Information

Innovative design of minimal invasive biodegradable poly(glycerol-dodecanoate) nucleus pulposus scaffold with function regeneration

Lizhen Wang[1#], Kaixiang Jin[1#], Nan Li[2], Peng Xu[1], Hao Yuan[1], Harsha Ramaraju[3], Scott J Hollister[3], Yubo Fan[1]*

[1]Key Laboratory of Biomechanics and Mechanobiology (Beihang University), Ministry of Education, Beijing Advanced Innovation Center for Biomedical Engineering, School of Biological Science and Medical Engineering, School of Engineering Medicine, Beihang University, Beijing, 100083, China.

[2] Department of Spine Surgery, Beijing Jishuitan Hospital, The Fourth Clinical Medical College of Peking University, Beijing, 100035, China.

[3]Wallace H. Coulter Department of Biomedical Engineering, Georgia Institute of Technology and Emory University, 313 Ferst Drive, Atlanta, GA 30332, USA

#These authors contributed equally: Lizhen Wang, Kaixiang Jin.

* Email: yubofan@buaa.edu.cn

COUNTENTS

Supplementary Figures 1 Thermo property of PGD polymer measured by DSC

Supplementary Figures 2 Tissue morphology and cell composition in NP/EP at 16 weeks

Supplementary Figures 3 Pathological section images of the disc with AF injury

Supplementary Figures 4 Pathological morphology of the disc with osteophyte

Supplementary Figures 5 Mechanical property of PGD at 20 and 37℃

Supplementary Figures 6 Fabrication and shape programing process of PGD NP scaffold

Supplementary Figures 7 Schematic of calculating disc height index

Supplementary Figures 8 Safranine O-stained images of the disc at 8 and 16 weeks

Supplementary Tables 1 Osteophyte incidence in the disc during 16 weeks implantation

Supplementary Tables 2 Histological grading scale of disk degeneration

Supplementary Tables 3 Histological scores at 8 and 16 weeks after implantation

Supplementary Tables 4 DHI and NP T2 MRI signal intensity during 16 weeks implantation

Supplementary Tables 5 Material parameters/elements types used in the numerical simulation

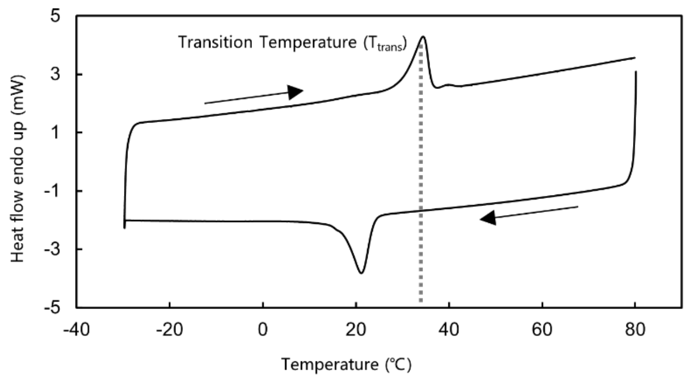

Supplementary Fig. 1 Thermo property of PGD measured by DSC. T_{trans} of PGD is set as endothermic peak in heating process.

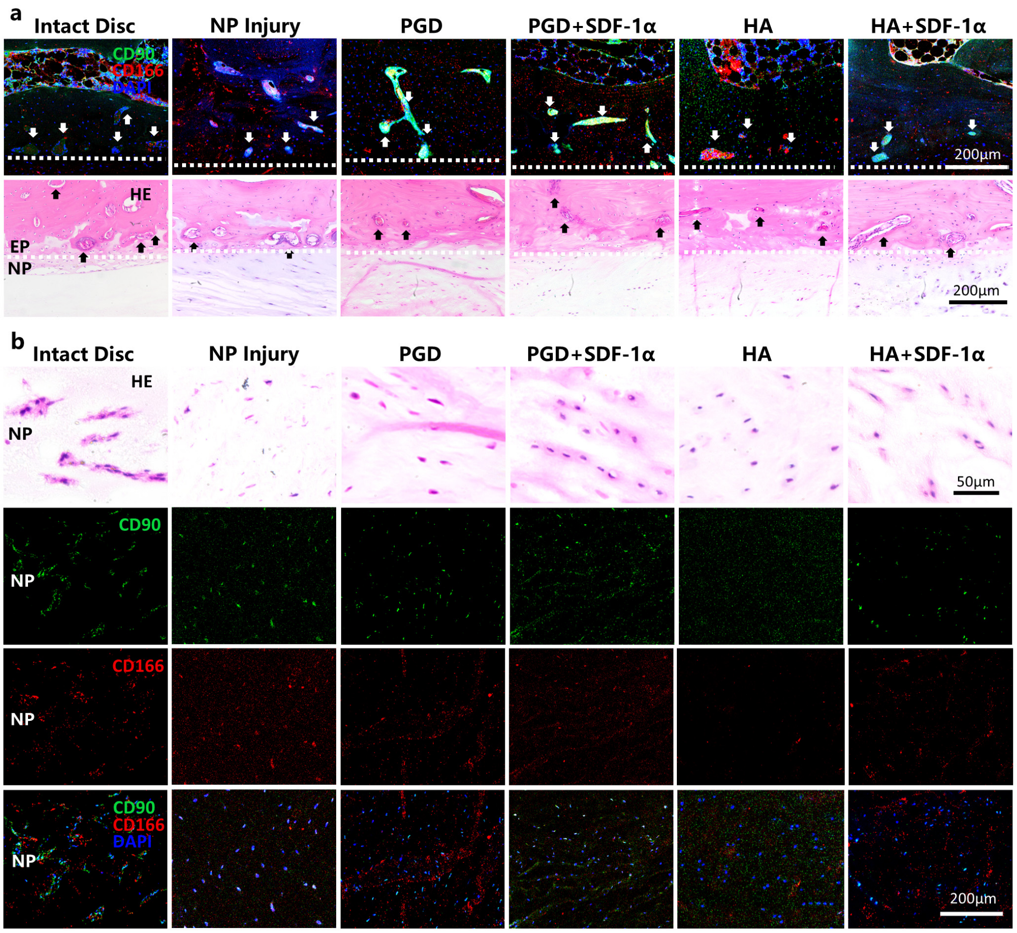

Supplementary Fig. 2 Tissue morphology and cell composition in NP/EP of Intact, NP Injury, PGD, PGD+SDF-1α, HA, and HA+ SDF-1α group. **a**, HE and immunofluorescence-stained histological images of the boundary between NP and EP at 16 weeks after implantation. White dotted line represents the boundary between EP and NP, and the black/white arrows indicate microvascular in EP. **b**, HE and immunofluorescence-stained histological images of NP at 16 weeks after implantation. Four times each group was repeated independently with similar results.

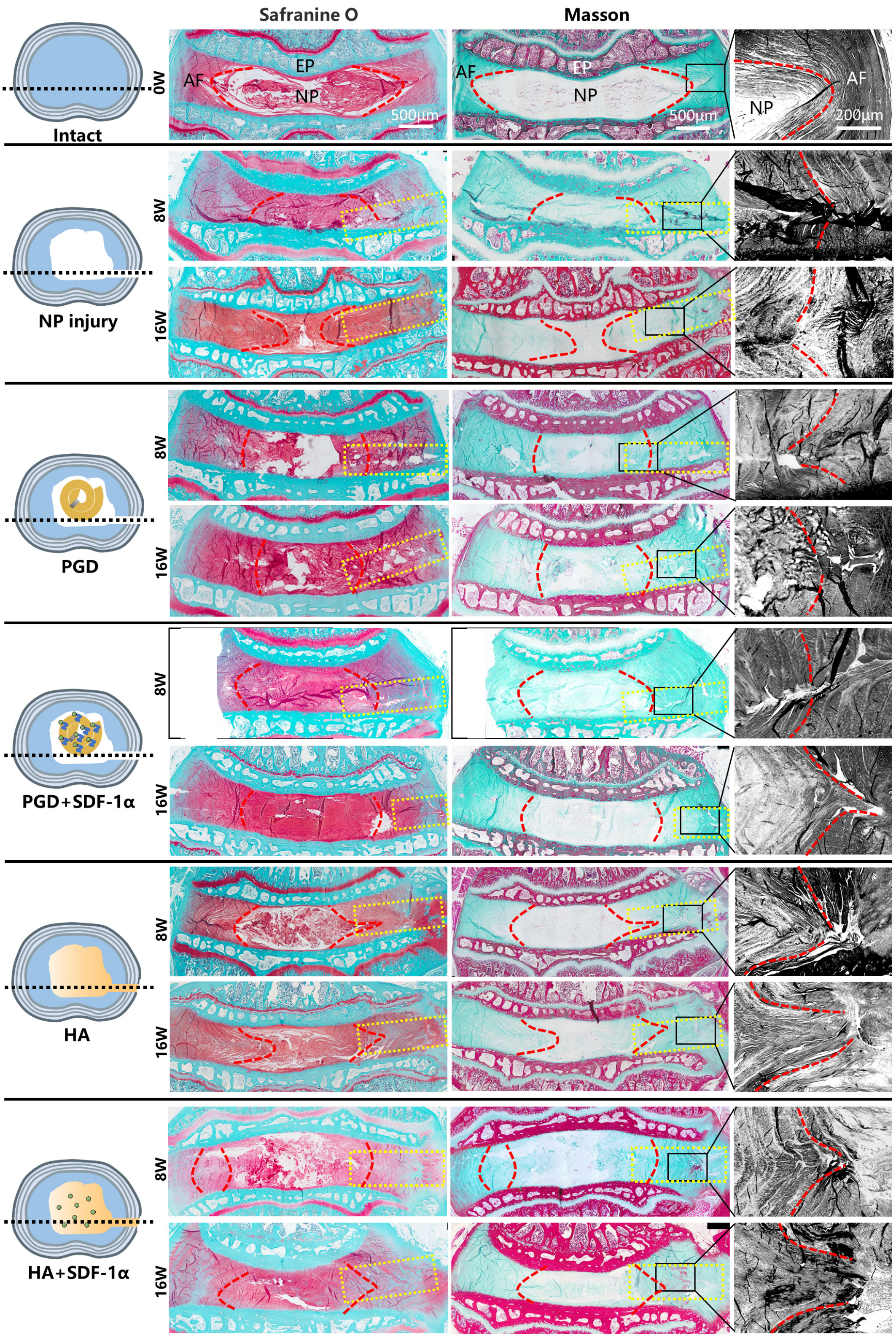

Supplementary Fig. 3 Pathological section images of the disc with AF injury. Safranine O and Masson-stained images of native intact disc, NP injury disc, and discs implanted with four NP scaffolds at 8 and 16 weeks. Yellow dotted box in the images represents the injury in AF. Red dotted lines represent AF outline of the disc. Four times each group was repeated independently with similar tissue morphology.

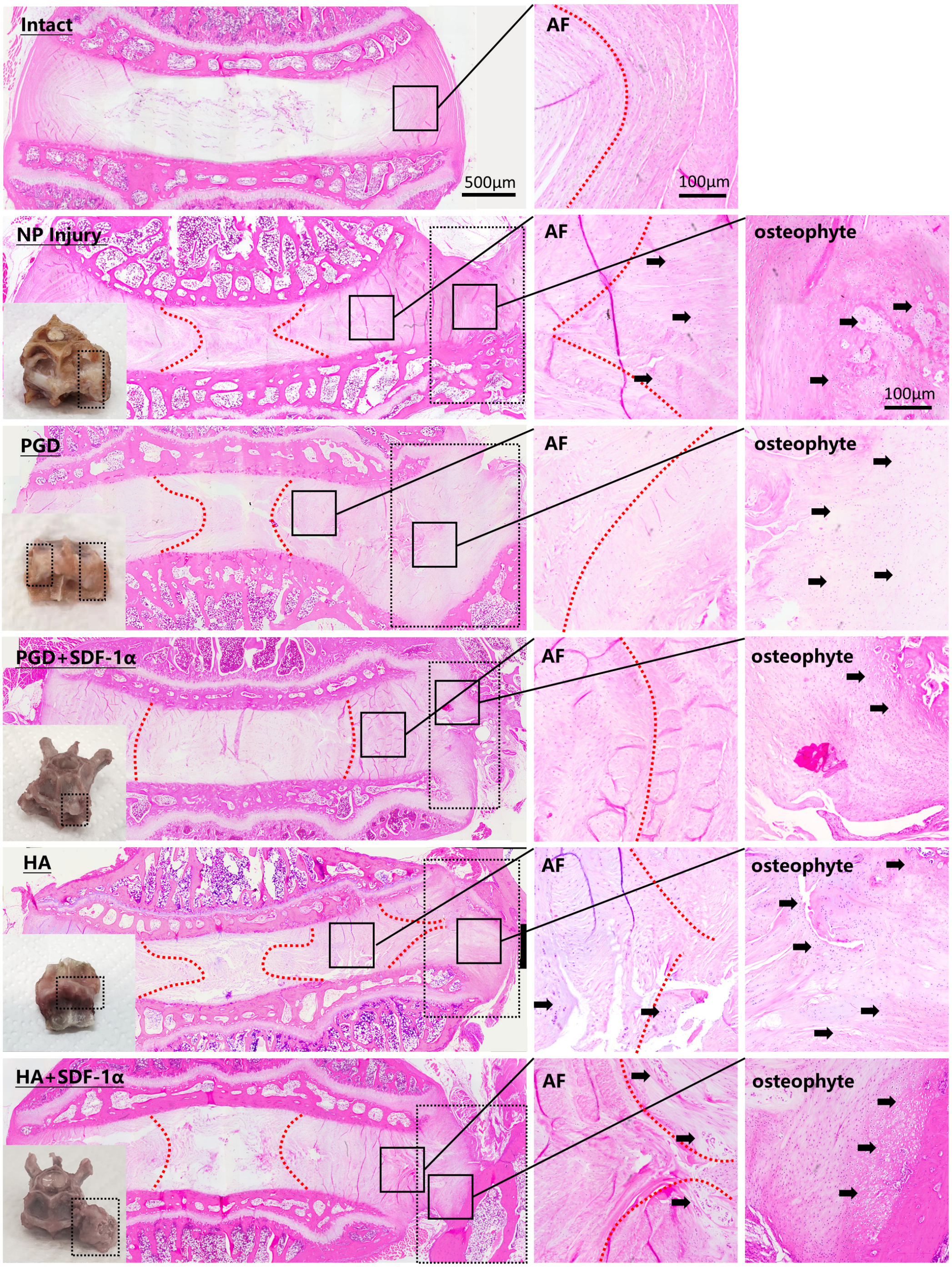

Supplementary Fig. 4 Pathological morphology of the disc with osteophyte in each experimental group. HE-stained images are taken from typical disc samples collected at 16 weeks after implantation, and AF / osteophyte are magnified to visualize their internal cellular distribution. Black dotted box indicates areas of osteophyte generated. Red dotted lines represent outline of AF in the disc. Black arrows indicate chondrocytes in AF/ osteophyte. Four samples each group was repeated independently, and osteophyte incidence was counted as shown in Supplementary Table 1.

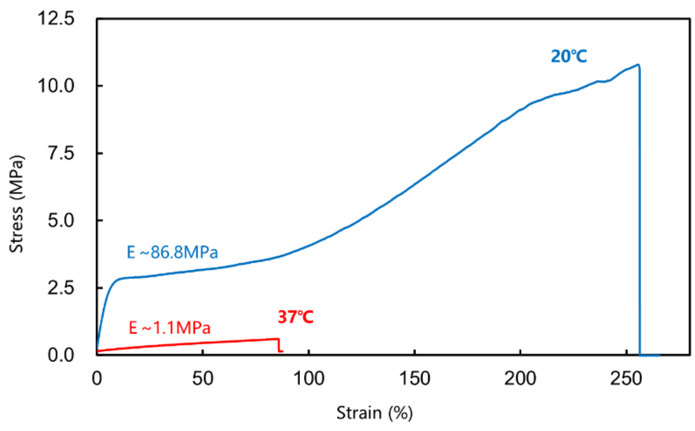

Supplementary Fig. 5 Mechanical property of PGD with optimized synthesis parameter ($MR_{H/C}$ =1.50 and t=72h) at room temperature and body temperature.

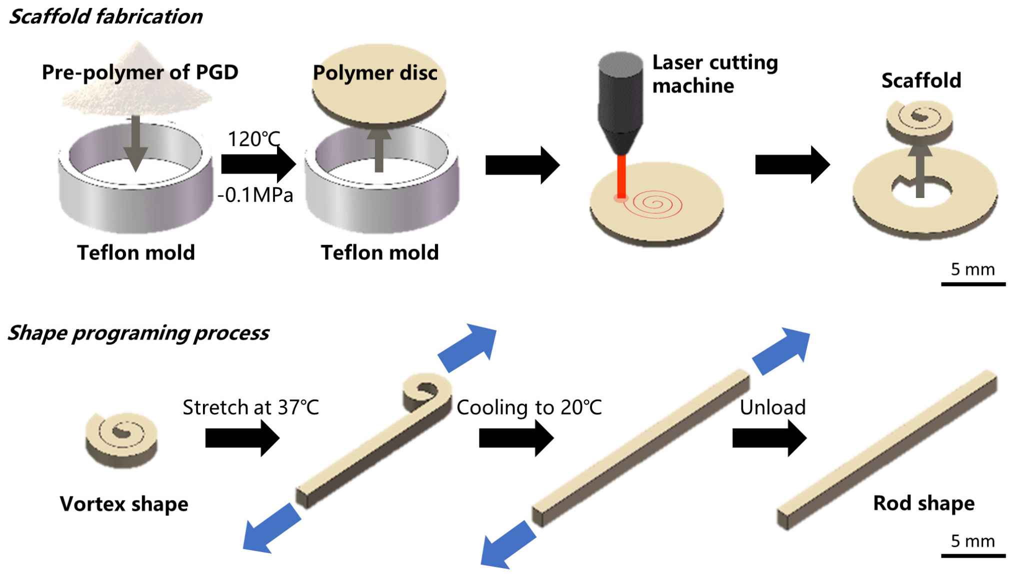

Supplementary Fig. 6 Fabrication and shape programing process of PGD NP scaffold with tendril structure.

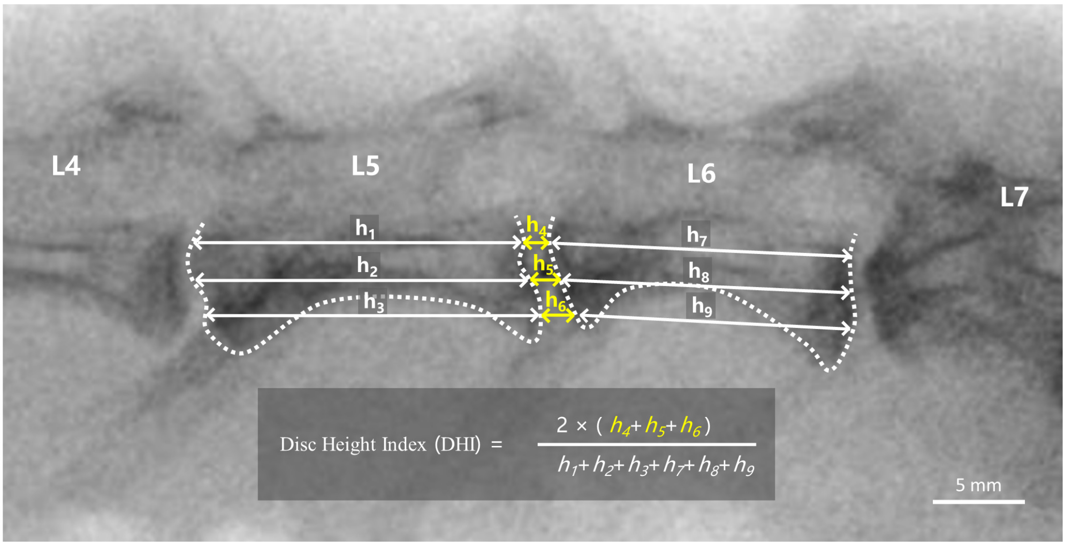

Supplementary Fig. 7 Schematic of calculating disc height index (DHI).

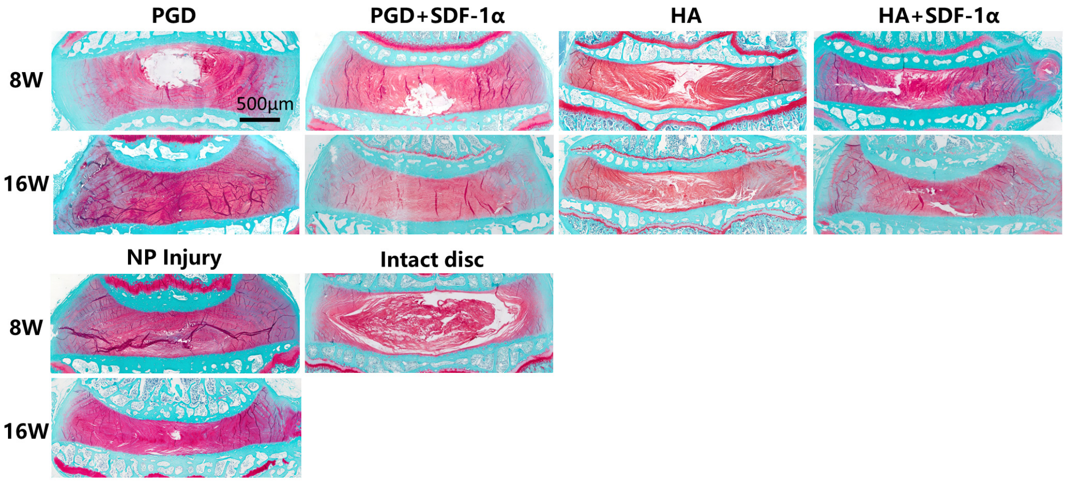

Supplementary Fig. 8 Safranine O-stained images of L5-L6 disc in the coronal plane at 8 and 16 weeks after implantation. Four times each group was repeated independently with similar morphology.

Groups	8 Week	16 Week	Total osteophyte incidence
Intact	0.0%	0.0%	0.0%
NP Injury	50.0% (2/4)	100.0% (4/4)	75.0% (6/8)
PGD	0.0% (0/4)	25.0% (1/4)	12.5% (1/8)
PGD+SDF-1α	0.0% (0/4)	25.0% (1/4)	12.5% (1/8)
HA	25.0% (1/4)	75.0% (3/4)	75.0% (6/8)
HA+SDF-1α	75.0% (3/4)	75.0% (3/4)	75.0% (6/8)

Supplementary Table 1. Osteophyte incidence in the intact, NP Injury, PGD, PGD+SDF-1α, HA, HA+SDF-1α groups at 8 and 16 weeks after implantation.

Cellularity and morphology	Grade
Cellularity of the annulus fibrosus	1. Fibroblasts comprise more than 75% of the cells<br>2. Neither fibroblasts nor chondrocytes comprise more than 75% of the cells<br>3. Chondrocytes comprise more than 75% of the cells
Morphology of the annulus fibrosus	1. Well-organized collagen lamellae without ruptured or serpentine fibers<br>2. Inward bulging, ruptured or serpentine fibers in less than one third of the annulus<br>3. Inward bulging, ruptured or serpentine fibers in more than one third of the annulus
Border between the annulus fibrosus and nucleus pulposus	1. Normal, without any interruption<br>2. Minimal interruption<br>3. Moderate or severe interruption
Cellularity of the nucleus pulposus	1. Normal cellularity with stellar-shaped nuclear cells evenly distributed throughout the nucleus<br>2. Slight decrease in the number of cells with some clustering<br>3. Moderate or severe decrease (> 50%) in the number of cells with all the remaining cells clustered and separated by dense area of proteoglycans
Morphology of the nucleus pulposus	1. Round, comprising at least half of the disc area in mid-sagittal sections<br>2. Rounded or irregularly shaped, comprising one quarter to half of the disc area in mid-sagittal sections<br>3. Irregularly shaped, comprising less than one quarter of the disc area in mid-sagittal sections

Supplementary Table 2. Histological grading scale of disk degeneration.

Histological scores		AF Cellularity (1-3)	AF Morphology (1-3)	AF-NP Border (1-3)	NP Cellularity (1-3)	NP Morphology (1-3)	Total Scores (1-15)
Intact		1.00±0.00	1.00±0.00	1.25±0.50	1.50±0.58	1.00±0.00	5.75±0.96
NP Injury	8 W	2.00±0.00	1.50±0.58	2.50±0.58	2.00±0.00	1.75±0.50	9.75±1.26
	16 W	2.25±0.50	2.75±0.50	2.75±0.50	2.00±0.00	2.75±0.50	12.50±1.29
PGD	8 W	1.25±0.50	2.25±0.50	2.00±0.00	2.00±0.00	1.75±0.50	9.25±0.96
	16 W	1.50±1.00	2.50±0.58	2.00±0.00	1.25±0.50	1.75±0.50	9.00±0.82
PGD+SDF-1α	8 W	1.25±0.50	2.25±0.50	1.75±0.50	1.50±0.58	1.75±0.50	8.25±0.51
	16 W	1.25±0.50	2.00±0.82	2.00±0.00	1.25±0.50	1.75±0.50	7.50±1.29
HA	8 W	1.50±1.00	2.25±0.50	2.00±0.82	3.00±0.00	2.00±0.82	10.75±1.71
	16 W	2.00±0.82	2.50±0.58	2.25±0.50	3.00±0.00	2.25±0.50	12.00±1.00
HA+SDF-1α	8 W	2.25±0.96	2.00±0.82	2.25±0.50	2.00±0.00	2.25±0.50	10.75±1.71
	16 W	1.25±0.50	1.75±0.96	2.50±0.58	2.75±0.50	2.25±0.50	10.50±1.00

Supplementary Table 3. Histological scores of each experimental group from AF cellularity, AF Morphology, AF-NP border, NP cellularity, and NP morphology parts.

Groups	1W		4W		8W		12W		16W	
	DHI (%)	MRI (%)	DHI (%)	MRI (%)	DHI (%)	MRI (%)	DHI (%)	MRI (%)	DHI (%)	MRI (%)
PGD	77.76± 6.53	54.08± 16.15	75.30± 7.52	50.46± 13.48	77.91± 9.98	48.32± 15.18	68.01± 5.88	45.33± 11.00	64.49± 4.77	45.75± 8.37
PGD+SDF-1α	86.63± 6.72	58.50± 16.36	82.70± 5.60	55.59± 11.07	81.86± 7.30	66.43± 9.94	85.16± 8.69	71.96± 13.51	85.46± 6.47	75.22± 9.92
HA	67.82± 2.60	63.42± 7.65	69.70± 9.77	62.21± 5.03	68.34± 7.26	57.41± 13.41	52.04± 8.97	65.03± 12.68	53.79± 4.29	51.71± 14.97
HA+SDF-1α	62.34± 9.70	57.33± 12.73	72.06± 8.49	72.06± 8.49	70.76± 8.22	64.46± 22.09	66.34± 4.64	65.67± 15.13	71.20± 5.16	71.42± 11.38
NP Injury	67.32± 4.72	61.46± 18.57	62.51± 10.80	53.40± 17.44	60.79± 6.33	47.38± 12.00	57.19± 5.85	47.31± 9.78	53.39± 4.73	45.33± 9.78

Supplementary Table 4. DHI and NP T2 MRI signal intensity of four experimental groups and NP Injury group during 16 weeks implantation.

Component	Young’s Modulus (MPa)	Poisson’s Ratio	Element Type
Cortical bone	10000	0.3	C3D8R
Trabecular bone	200	0.315	C3D8R
Endplate	23.8	0.4	C3D8H
Nucleus pulposus	1	0.499	C3D8H
Annular ground substance	3.4	0.4	C3D8H
Annulus fiber	500	0.35	SFM3D4
PGD *	1.163	0.47	C3D8H
HA	0.00697	0.48	C3D8H

Supplementary Table 5. Material parameters and elements types used in the numerical simulation.
