# Supplementary material for: Innovative design of minimal invasive biodegradable poly(glycerol-dodecanoate) nucleus pulposus scaffold with function regeneration
Source: Nat Commun. 2023 Jun 30;14:3865. doi: 10.1038/s41467-023-39604-0 (PMC10313828; doi:10.1038/s41467-023-39604-0)
Supplement: Supplementary file 3 — Description of Additional Supplementary Files [file 41467_2023_39604_MOESM3_ESM.pdf]

Description of Additional Supplementary Files

File Name: Supplementary Movie 1
Description: Shape transformation of PGD NP scaffold at 37℃ water.

File Name: Supplementary Movie 2
Description: Shape transformation of PGD NP scaffold at 37℃ air.

File Name: Supplementary Movie 3
Description: Finite element analysis of rabbit L5-L6 lumbar disc model with HA NP scaffold.

File Name: Supplementary Movie 4
Description: Finite element analysis of rabbit L5-L6 lumbar disc model with PGD NP scaffold.

File Name: Supplementary Movie 5
Description: Shape programing of PGD NP scaffold before implantation.

File Name: Supplementary Data 1
Description: Number of microvascular in EP at 8 and 16 weeks.

File Name: Supplementary Data 2
Description: Percentage of CD90 and CD166 positive cells in NP at 8 and 16 weeks.

File Name: Supplementary Data 3
Description: Histological scores of disc degeneration for native intact disc, NP injury disc and four experimental groups at 8 and 16 weeks.

File Name: Supplementary Data 4
Description: Normalized DHI variation of L5-L6 disc in NP injury disc and four experimental groups during 16 weeks implantation.

File Name: Supplementary Data 5
Description: Normalized T2 signal intensity variation of L5-L6 disc in nucleus cavity of NP injury disc and four experimental groups during 16 weeks implantation.

File Name: Supplementary Data 6
Description: Effective instantaneous and equilibrium moduli of the disc at 16 weeks.
