# Supplementary material for: Innovative design of minimal invasive biodegradable poly(glycerol-dodecanoate) nucleus pulposus scaffold with function regeneration
Source: Nat Commun. 2023 Jun 30;14:3865. doi: 10.1038/s41467-023-39604-0 (PMC10313828; doi:10.1038/s41467-023-39604-0)
Supplement: Supplementary file 15 — Reporting Summary [file 41467_2023_39604_MOESM15_ESM.pdf]

nature portfolio

Corresponding author(s): Yubo Fan

Last updated by author(s): May 30, 2023

Reporting Summary

Nature Portfolio wishes to improve the reproducibility of the work that we publish. This form provides structure for consistency and transparency in reporting. For further information on Nature Portfolio policies, see our Editorial Policies and the Editorial Policy Checklist.

Statistics

For all statistical analyses, confirm that the following items are present in the figure legend, table legend, main text, or Methods section.

n/a	Confirmed	
☐	☒	The exact sample size (*n*) for each experimental group/condition, given as a discrete number and unit of measurement
☐	☒	A statement on whether measurements were taken from distinct samples or whether the same sample was measured repeatedly
☐	☒	The statistical test(s) used AND whether they are one- or two-sided<br>*Only common tests should be described solely by name; describe more complex techniques in the Methods section.*
☐	☒	A description of all covariates tested
☐	☒	A description of any assumptions or corrections, such as tests of normality and adjustment for multiple comparisons
☐	☒	A full description of the statistical parameters including central tendency (e.g. means) or other basic estimates (e.g. regression coefficient) AND variation (e.g. standard deviation) or associated estimates of uncertainty (e.g. confidence intervals)
☐	☒	For null hypothesis testing, the test statistic (e.g. *F*, *t*, *r*) with confidence intervals, effect sizes, degrees of freedom and *P* value noted<br>*Give P values as exact values whenever suitable.*
☒	☐	For Bayesian analysis, information on the choice of priors and Markov chain Monte Carlo settings
☒	☐	For hierarchical and complex designs, identification of the appropriate level for tests and full reporting of outcomes
☒	☐	Estimates of effect sizes (e.g. Cohen's *d*, Pearson's *r*), indicating how they were calculated

Our web collection on statistics for biologists contains articles on many of the points above.

Software and code

Policy information about availability of computer code

Data collection	Commercial softwares used to collect the data in this study are the following: Pyris software (version 5.0, Platinum Elmer) for DSC analysis, Leica LAS X(version 3.7.4.23463, Leica) for confocal analysis, SkanIt (version 7.0, ThermoFisher) for colorimetric analysis, cellSens (version 1.7, OLYMPUS), ABAQUS (version 6.14, Dassault Systèmes) for numerical simulation.
Data analysis	Commercial softwares used to analysis the data in this study are the following: SPSS Statistics (version22, IBM), Excel (version 2019, Microsoft), DicomGo (version 2.0.1.4, Link Imaging), ImageJ (version 1.6.0, NIH).

For manuscripts utilizing custom algorithms or software that are central to the research but not yet described in published literature, software must be made available to editors and reviewers. We strongly encourage code deposition in a community repository (e.g. GitHub). See the Nature Portfolio guidelines for submitting code & software for further information.

Data

Policy information about availability of data

All manuscripts must include a data availability statement. This statement should provide the following information, where applicable:

- Accession codes, unique identifiers, or web links for publicly available datasets
- A description of any restrictions on data availability
- For clinical datasets or third party data, please ensure that the statement adheres to our policy

Data Availability section have been added in the manuscript. Supplementary Data 1-6 have been provided to show experimental data and statistical analysis

generated in this study, including histological analysis, microvascular in EP, MSCs proportion in NP, DHI variation during 16 weeks implantation, MRI T2 signal intensity variation during 16 weeks implantation, and disc mechanical properties after implantation.

Human research participants

Policy information about studies involving human research participants and Sex and Gender in Research.

Reporting on sex and gender	n/a
Population characteristics	n/a
Recruitment	n/a
Ethics oversight	n/a

Note that full information on the approval of the study protocol must also be provided in the manuscript.

Field-specific reporting

Please select the one below that is the best fit for your research. If you are not sure, read the appropriate sections before making your selection.

☒ Life sciences ☐ Behavioural & social sciences ☐ Ecological, evolutionary & environmental sciences

For a reference copy of the document with all sections, see nature.com/documents/nr-reporting-summary-flat.pdf

Life sciences study design

All studies must disclose on these points even when the disclosure is negative.

Sample size	At least 4 independent repeats for each experiment in this study have been used to reflect the experimental results objectively.
Data exclusions	No data were excluded from the analyses.
Replication	Quantified tests applied in this study were performed using at least 4 independent repeats for each experimental group. The reproducibility of our results was displayed by the data-points in the Figures, and source data and statistical analysis were all available in the Supplementary Data.
Randomization	All experiment sample/animal in this study were allocated randomly. Polymer samples of each experimental group were synthesized before implantation and randomly selected for grouping. Experimental animals were also picked randomly from healthy animals with fitted weight and age before implant surgery for each experimental group.
Blinding	The investigators were blinded to group allocation during data collection and analysis.

Reporting for specific materials, systems and methods

We require information from authors about some types of materials, experimental systems and methods used in many studies. Here, indicate whether each material, system or method listed is relevant to your study. If you are not sure if a list item applies to your research, read the appropriate section before selecting a response.

Materials & experimental systems

n/a	Involved in the study	
☐	☒	Antibodies
☐	☒	Eukaryotic cell lines
☒	☐	Palaeontology and archaeology
☐	☒	Animals and other organisms
☒	☐	Clinical data
☒	☐	Dual use research of concern

Methods

n/a	Involved in the study	
☒	☐	ChIP-seq
☒	☐	Flow cytometry
☒	☐	MRI-based neuroimaging

Antibodies

Antibodies used	Primary antibodies: Anti-CD90 (ab225, abcam); Anti-CD166 (ab235957, abcam);

Antibodies used	Secondary antibodies: Alexa-fluor 488-labeled anti-mouse antibody (A32723); Alexa-fluor 647-labeled anti-rabbit antibody (A32733).
Validation	Validation was provided through the manufacturer's product manual and the published research article use/validation of the relevant antibodies by other research groups listed in the manufacturer's website (abcam.com, eg.Eur J Med Res 27:5 (2022) for CD90 and NPJ Regen Med 6:26 (2021) for CD166.).

Eukaryotic cell lines

Policy information about cell lines and Sex and Gender in Research

Cell line source(s)	BALB/C 3T3 cells used in this study were commercially obtained from National BioMedical Cell-Line Resource (Peking Union Medical College, China).
Authentication	Authentication was provided by National BioMedical Cell-Line Resource (Peking Union Medical College, China).
Mycoplasma contamination	Cells used in this study were confirmed negative for mycoplasma contamination from the supplier.
Commonly misidentified lines (See ICLAC register)	No commonly misidentified cell lines were used in the study.

Animals and other research organisms

Policy information about studies involving animals; ARRIVE guidelines recommended for reporting animal research, and Sex and Gender in Research

Laboratory animals	Sprague-Dawley rats weighing 250-300g at 6-8 weeks were used to subcutaneous implant polymer samples. New Zealand white rabbits weighing 2.5-3.5kg at 6-8 months of age were selected as experimental animals to implant the nucleus pulposus scaffolds.
Wild animals	No wild animals were used in the study.
Reporting on sex	Sex was not considered in this study design.
Field-collected samples	No field collected samples were used in the study.
Ethics oversight	In-vivo experimental procedures were performed following the Beihang University’s Committee on Use and Care of Animals (Permit No.BM201900084 for rats' experiments, No.BM20200146 for rabbits' experiments).

Note that full information on the approval of the study protocol must also be provided in the manuscript.
